# Variations in Postprandial Blood Glucose Responses and Satiety after Intake of Three Types of Bread

**DOI:** 10.1155/2011/437587

**Published:** 2011-05-31

**Authors:** Marianne S. H. Lunde, Victoria T. Hjellset, Gerd Holmboe-Ottesen, Arne T. Høstmark

**Affiliations:** Section of Preventive Medicine and Epidemiology, Institute of Health and Society, University of Oslo, P.O. Box 1130 Blindern, 0318 Oslo, Norway

## Abstract

*Background*. The magnitude and duration of postprandial blood glucose (PPG) elevations are important risk factors of diabetes and coronary heart diseases. *Aim*. To study PPG after ingestion of breads with and without pea fibre and rapeseed oil. *Methods*. After fasting overnight, 10 Pakistani immigrant women participated in three experiments having a crossover design and involving ingestion of various types of bread: regular coarse bread or fibre enriched-bread with two levels of rapeseed oil, all providing 25 g available carbohydrates (CHO). Blood glucose and satiety were determined before the meal and every 15 min over the next 2 hours. *Results*. Intake of an amount of pea fibre-enriched bread containing 25 g CHO attenuated, the postprandial peak glucose value, the incremental area under the glucose versus time curve during 15 to 75 min, and the glycemic profile, and increased duration of satiety (*P* < .05), as compared with intake of regular bread with 25 g carbohydrate. *Conclusion*. Pea fibre-enriched breads can reduce PPG and prolong satiety.

## 1. Background

The burden of cardiovascular diseases (CVD), obesity, and diabetes is rapidly increasing worldwide [1–3]. Disturbances in blood glucose levels are implicated in the development of these diseases [4]. Normally, the blood glucose concentration is well regulated by hormones, such as insulin and several counterregulatory hormones, but many external factors such as physical activity [5], emotional status, [6] and diet [7] will also modify the blood glucose levels. 

In particular, the postprandial blood glucose concentration appears to play a critical role in progression of diabetes and CVD [8, 9]. In a meta-analysis of observational studies from 2008, Barclay et al. [10] found that postprandial hyperglycemia contributes to chronic disease, independently of diabetes status. Benefits of reducing PPG levels have been demonstrated in connection with chronic diseases in general [11] and for CVD [9, 12], diabetes, and obesity [4, 13] in particular. It seems accordingly of special importance to avoid sustained hyperglycaemic episodes and large blood glucose fluctuations during the day. Since the highest glucose levels occur in the postprandial period, glycaemic control in this period is important, especially after ingestion of high glycaemic foods (e.g., certain types of bread). 

Postprandial blood glucose is largely influenced by the carbohydrate load of the meal implying that diets with low glycemic loads are beneficial in controlling postmeal plasma glucose [7]. Several studies have suggested that the blood glucose fluctuations are associated with oxidative stress and inflammation [14–16]. The term glycemic profile (GP) has been introduced defined as the duration of the incremental postprandial blood glucose response divided with the blood glucose incremental peak (min/mM) [17]. 

GL of a meal can be lowered either by reducing amount ingested and/or by reducing the GI of the meal. The latter can for example be achieved by using fibre-enriched foods [7]. It is well known that both the type and amount of carbohydrates can modify the postprandial glucose levels [18]. Fibre is often categorized as soluble or insoluble ones. Intake of soluble fibre may result in formation of health-promoting compounds during fermentation in the large bowel whereas insoluble fibres increases and softens the stool bulk, thereby shortening the transit time through the intestinal tract [19]. In addition, fibre may have the ability to bind bile acids and decrease reabsorption of bile acids and cholesterol from the intestine. 

Since dietary fibre seems to have a beneficial influence on glucose homeostasis, it is generally recommended that people should be encouraged to increase their fibre consumption, for example, from whole grains [20]. However, inclusion of fibre and whole grains seem to complicate the industrial production of bread.

Pea fibre has traditionally not been a common ingredient in bread recipes. It is, however, known that intake of whole peas will influence blood glucose level [7]. Accordingly, replacing available carbohydrates in breads by pea fibre may potentially reduce the glycemic impact of such breads. In 2009, Marinangeli et al. concluded that whole yellow-pea flour can be used to produce low glycemic functional foods possessing sensory attributes that are comparable to identical food products containing whole-wheat flour [21]. The baking industry in Norway has recently succeeded in making pea fibre-enriched bread without compromising the bread production methods.

There are large intraindividual differences in the population in regard to postprandial levels of blood glucose after intake of carbohydrates. Progression towards type 2 diabetes manifests as a gradual deterioration of postprandial blood glucose control. These differences in blood glucose are most pronounced after ingestion of food that has the potential to increase blood glucose the most, that is, a meal with high glycemic load. There may be ethnic differences in the glycemic response to carbohydrates. Dickinson et. al [16] found that the postprandial hyperglycemia and insulin sensitivity varied among different ethnic groups, and that lean, young South East Asians had the highest postprandial glycemia and the lowest insulin sensitivity in response to a realistic carbohydrate load. 

The first aim of the present work was to examine to what extent the PPG response might be attenuated by replacing some of the digestible carbohydrates with pea fibre. When comparing PPG responses to these breads, great care was taken to ensure that equal quantities of available carbohydrates were ingested, but different amount of fibre. Secondly, since ingestion of fat with carbohydrates has previously been reported to favorably reduce PPG [22, 23], we also studied whether inclusion of rapeseed oil to the fibre-enriched bread recipe would influence the PPG response. Again, great care was taken to ensure that the ingested amount of available carbohydrates were similar in breads with different amounts of rapeseed oil. Thirdly, we wanted to evaluate the satiety after ingestion of the different types of bread.

## 2. Methods

### 2.1. Ethics

The study was carried out according to the Helsinki declaration and was approved by The National Committees for Research Ethics, Norway, 2.2008.2456. Participants gave informed and written consent.

### 2.2. Subjects

The 10 participants were recruited from the InnvaDiab-DEPLAN lifestyle intervention study among female Pakistani immigrants living in Oslo with a high risk of developing type 2 diabetes [24]. Five participants had impaired glucose tolerance following an oral glucose tolerance test (OGTT) with blood glucose concentration above 7.8 mmol/L 2 h after glucose ingestion. Baseline data for the participating subjects are presented in Table 1. Mean age of the participants was 46 years (range: 30 to 59), and mean body mass index (BMI; kg/m^2^) 31.0 (range: 25.7 to 41.9). The average glycated hemoglobin (HbA1_c_) value was 5.6% (range: 4.9 to 6.1). Fasting blood glucose measured on the three experimental days was 5.6 (range: 4.7 to 6.7). All participants had waist circumference ≥80 cm. These immigrants have adapted to Western eating habits [25], of which Norwegian leavened whole-wheat bread is a major component. Subjects on glucose-lowering medication were excluded from the study.

### 2.3. The Breads

Three types of bread were tested in this study, and all were baked, cooled, and frozen according to a standardized procedure at Idun Industries (Idun, Norway), and the baking process was the same for all breads. The physical characteristics, that is, colour, crust firmness, specific volume, and the sensory properties of the breads were kept as similar as possible.

The content of available carbohydrates, total starch, and dietary fibre was provided by the manufacturer, and based upon calculations. 

All the loaves were for the most part based on wheat flour, and for the fibre-enriched breads the main fibre source used was pea hull fibre. The chemical profile of the pea hull fibre is 85–90% of dietary fibre (approx. 1/6 of soluble/insoluble fibre), 10–15% of protein/starch/fat/oligosaccharides, and simple sugars.

The content of bread was as follows in falling order.

**Regular Coarse Bread, Bread 1 (Low Fibre, Low Fat)**

Water, wholemeal wheat flour, wheat flour, yeast, margarine (vegetable oil), water, salt, hydrogenated vegetable fat, emulsifier (E471), acidity regulator (E330), flavour, salt, wheat gluten, wholemeal rye, dried sourdough of wheat, rapeseed oil, and enzymes.

**Fibre-Enriched Bread, Bread 2 (High Fibre, Low Fat)**

Water, wheat flour, pea fibre, wheat gluten, barley, yeast, dried sourdough from wheat, salt, alginate, pectin, dextrose, oat, dried sourdough from rye, emulsifier (E472e), ground whole rye, rapeseed oil, ascorbic acid (E300), and enzymes.

**Fibre-Enriched Bread, Bread 3 (High Fibre, High Fat)**

Water, wheat flour, pea fibre, rapeseed oil, wheat gluten, yeast, dried sourdough from wheat, salt, alginate, pectin, dextrose, oat, dried sourdough from rye, emulsifier (E472e), ground whole rye, ascorbic acid (E300), and enzymes.

The breads varied in content of energy, macronutrients, and dietary fiber.

Bread 1, per 100 g: 220 kcal, 4.6 g fibre, 8.7 g protein, 40.1 g CHO (minus fibre), and 2.8 g fat.
Bread 2, per 100 g: 145 kcal, 17 g fibre, 12 g protein, 21 g CHO (minus fibre), and 1 g fat.
Bread 3, per 100 g: 200 kcal, 17 g fibre, 11 g protein, 19 g CHO (minus fibre), and 9 g fat.

### 2.4. The Meals

Sliced bread, if preferred toasted, was served with 200 mL of tap water. We calculated portion sizes so as to obtain 25 g available carbohydrate. Data for macronutrient composition, including available carbohydrate and dietary fiber, was provided by the manufacturer. The portion sizes are shown in Table 2. Great care was taken to ensure that equal quantities of available carbohydrates were ingested.

### 2.5. Experimental Procedures

On separate days and after an overnight fast, 10 subjects participated in three experiments, in a randomized crossover design. On experimental days, the subjects arrived at 0845 hrs and sat resting until 0900 hrs. At that point, the fasting blood glucose concentration was measured in triplicate, and subsequently one test meal of bread was served. The subjects consumed the bread in a comfortable place within 15 min. The postmeal blood glucose concentration was measured repeatedly during a 2-hour period after the start of the test meal. The test meals were consumed in random order on separate mornings with one week between subsequent sessions.

### 2.6. Anthropometric and Blood Measurement Data

Body weight was measured on the last experimental day. Data for age, height, waist circumference, blood pressure, HbA1_c_, and OGTT were collected on a separate day as part of the InnvaDiab-DEPLAN study [24] in which the subjects had participated.

### 2.7. Pre- and Postprandial Blood Sampling and Blood Glucose Measurements

Capillary blood glucose concentrations (mmol/L) were measured (Ascensia Contour, Bayer) before the meal and again at 15, 30, 45, 60, 75, 90, 105, and 120 min in the postprandial phase. As informed by the manufacturer, the glucometers from Bayer had an accuracy of ≤10% thus providing clinically and analytically acceptable results [26, 27]. The blood glucose concentration before each meal (time = 0) was measured ×3, and the average value was used as the basal value in the statistical calculations. The same apparatus was used to measure blood glucose on each participant all test days.

### 2.8. Satiety

Immediately after each blood sample was collected, the subjects rated their subjective feeling of satiety using a 7-point category rating scale where 1 is very hungry and 7 is no hunger at all. Ratings were completed shortly after the blood samples were obtained.

### 2.9. Statistical Methods

The primary outcome of this study was the overall postprandial glycemia, as estimated by several measures:

PV: postprandial blood glucose peak value,
TTP: time to reach PV from zero time,
IPV: incremental PV, that is, PV minus fasting blood glucose,
GP: glycemic profile, that is, the duration of the incremental postprandial blood glucose response divided with the blood glucose incremental peak,
IAUC: the incremental area under the glucose versus time curve, calculated by the linear trapezoidal rule (WHO 1997).

Data was analyzed using SPSS 15.0. All data were assessed for normal distribution of values. Comparison of mean values of normally distributed data within groups were performed by paired samples Student's *t*-test. For each group, a two-factor within-subject repeated measure ANOVA was used to test the effects of time and type of bread, and the interaction between time and type. Data are reported as mean values ± SEM in figures, or mean with SD in text and tables. When appropriate, the results were adjusted for baseline values.

## 3. Results

The fasting blood glucose level was 5.6 ± 0.6 mmol/L all three test days.

### 3.1. Postprandial Blood Glucose Response to Different Types of Bread

**Figure 1:**
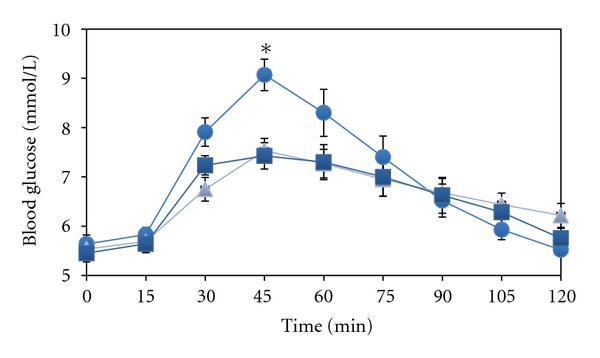
Blood glucose concentration as influenced by intake of various types of bread. On separate days and after an overnight fast, the blood glucose concentration was determined after ingestion of three types of bread which were given in portions providing 25 g carbohydrates, *n* = 10. Total weight of the breads varied, being 61 g for Bread 1, which is regular coarse bread (closed circles), 119 g for Bread 2 which is bread with 17% pea fibre and 1% rapeseed oil (squares), and 133 g for Bread 3 containing 17% pea fibre and 9% rapeseed oil (triangles). Mean values ± SEM. Note broken *y*-axis. *P < .05 versus corresponding value when ingesting pea fibre-enriched bread.

In response to ingesting 25 g CHO from Bread 1 the blood glucose concentration increased gradually and reached a peak value of 9.1 mmol/L after 45 min (Figure 1). Then there was a gradual decrease to the initial value after 120 min. Average blood glucose curves had the same qualitative time course after ingestion of fibre-enriched bread, but the postprandial glucose excursions, estimated by PV, IPV, and GP, were attenuated by Bread 2 and 3 as compared with Bread 1 (*P* < .05). Considering the whole 120 min experiment, there were no significant group differences in IAUC. However, IAUC in the time period 15 to 75 min after ingestion of bread was significantly reduced after ingestion of pea fibre-enriched bread (*P* < .05). There was no difference between Bread 2 and 3 in PV, IPV, and IAUC, Table 3.

Using repeated measures ANOVA, we found a significant main effect of time and type of bread (*F* = 30.7 and 24.7, resp., *P*-value < .001) and an interaction between time and type (*P* < .005).

### 3.2. Satiety

**Figure 2:**
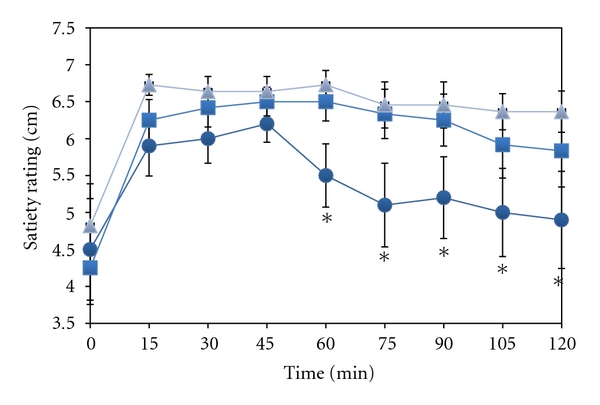
Satiety rating in the time period after intake of various amounts and types of bread. On separate days and after an overnight fast, 10 subjects rated their satiety during two hours after ingestion of various amounts and types of bread: triangles = pea fibre-enriched bread containing 25 g carbohydrate with 9% rapeseed oil; squares = pea fibre-enriched bread containing 25 g carbohydrate and 1% rapeseed oil; circles = regular coarse bread containing 25 g carbohydrate. Satiety was rated on a 7-point scale where 1 is very hungry and 7 completely satisfied. Mean values ± SEM. Note broken *y*-axis. *P < .05 versus corresponding value when ingesting same amount of available CHO as pea fibre-enriched bread.

For all experiments, the average zero point satiety was rated between 4 and 5 and increased as the participants consumed the bread during the first 15 min (Figure 2). The satiety levelled off after 15 min and was sustained during the trial period after ingestion of 25 g CHO as fibre-enriched bread with 9% fat. The mean satiety decreased 45 min after intake of 25 g CHO ingested in regular coarse bread. By contrast, only a slight decrease in satiety was observed even 75 min after intake of 25 g CHO ingested in fibre-enriched bread with 1% fat. The subjectively rated satiety values after intake of 25 g CHO in regular coarse bread (bread 1) was significantly different from the other test meals at all time points from 60 min and throughout the observation period (*P* < .05 for all time points). Corrections for variations in baseline values did not change the outcome. It seems accordingly that under the conditions of this study the amount of ingested bread, rather than amount of carbohydrates or energy governs the level of satiety.

## 4. Discussion

Previous studies have shown that fibre can lower postprandial glycemia [28, 29]. It seems, however, difficult to make acceptable breads containing high amounts of fibre, but pea fibre enrichment seems to be an exception. The present study demonstrates that replacing some of the digestible carbohydrates in bread with pea fibre can appreciably reduce blood glucose elevation after intake of such bread. Therefore, regular intake of pea fibre-enriched breads could be a useful approach to counteract high excursions of blood glucose. Additionally, inclusion of pea fibre seems to allow the subjects to eat a satisfying quantity of bread, maintaining satiety high over a longer period, and at the same time keeping the postprandial blood glucose levels significantly lower than after intake of the control bread. The participants of this experiment were not asked if they found the breads palatable, but none of them complained about ingesting the breads. Additionally, in a separate, yet unpublished, study the palatability of the pea fibre-enriched breads was reported to be good. Inclusion of pea fibre-enriched bread would represent a small change in the diet as the appearance of the bread is similar to other industrial baked regular coarse bread and do not represent a major change in eating habits as often seen for more special diets. 

We emphasize that our data are not sufficient to conclude that it is the pea fibre *per se* which causes the reduced postprandial glycemia. Thus, the control bread and the pea fibre bread contained different ingredients other than just the pea fibre. For example, the regular bread contained wheat flour and whole meal wheat flour whereas the pea fibre-enriched bread contained wheat flour and ground whole rye. Rye tends to produce lower PPG with respect to wheat and the coarseness of the grind can also have an effect [30]. Additionally, the control bread contained dried sourdough of wheat whereas the pea fibre bread contained dried sourdough of wheat and dried sourdough from rye. Rye, coarseness of grind, and sourdough have been found to lower PPG in various studies [31–33]. Thus, differences in postmeal glycemia (or satiety) between the breads could partly be attributed to these differences. Nevertheless, the major difference between the breads was the pea fibre content. We therefore have used the term “pea fibre-enriched bread” as a descriptive term of bread containing a great percentage of pea fibre, with no allusion to a causal effect of pea fiber per se. It is widely accepted that repeated, high postprandial levels of blood glucose have a negative impact on health. The harmful effects of postprandial hyperglycemia may partly be related to the production of free radicals. As shown by Ceriello et al., intake of a carbohydrate meal is followed by oxidative stress that is related to the level of hyperglycemia [34], and to fluctuations in blood glucose levels [14, 16, 35]. In our study we did not measure free radicals. It is therefore impossible to assess the influence of pea fibre-enriched bread on these variables, but it is tempting to speculate if the appreciable attenuating effect upon PPG could reduce free radical production. 

The incremental area under the glucose versus time curve (IAUC) is probably an appropriate indicator of the glycation potential. In this work we did find a significant reduction of IAUC in the time period 15–75 min after intake of the pea fibre-enriched bread as compared with control. However, the IAUC for the entire 2-hour period did not differ significantly between the experiments. In this context there might have been a power problem, and the possibility of making a Type 2 error should be considered. However, coingestion of pea fibre attenuated the postprandial blood glucose response as estimated by PV, and hence presumably also lowered the glycation potential. Vuksan et al. have recently reported reductions in IAUC as a result of coingestion of whole grain (Salvia Hispanica L.) baked into with bread [36]. 

To reduce body weight, lowered caloric intake has been prescribed. There is, however, a problem with hunger when using low-energy diets. Measurements of complex sensations such as hunger is difficult and one concern with categorical rating scales is that the lack of clarity on how to understand the questions and how to report complex sensations [37]. Nevertheless, our data show that addition of pea fibre to bread resulted in both low PPG and a higher satiety rating. Thus, this could seem to offer an alternative approach to counteract overweight and possibly insulin resistance. Further studies are, however, required to corroborate this hypothesis. In favour of the hypothesis is a recent study in which it was concluded that reducing the glycemic load of a meal by lowering the glycemic index seemed an effective strategy to increase energy expenditure after a meal [38]. 

There might be great differences in digestibility and absorbability of different types of carbohydrate-rich foods, resulting in great variation in their PPG effects. The present results strongly suggest that high PPG and large blood glucose fluctuations during the day can be counteracted by using pea enriched bread instead of the regular types of coarse bread baked with more than 50% wholegrain without any additional fibre sources.

The use of carbohydrate-rich foods manifests differently in different cultures around the world. Nevertheless, inclusion of such foods prepared according to different local recipes is common. Refined high GI products are often cheap, easily accessible, and palatable. An important public health strategy could be to increase the number of available processed carbohydrate-rich foods with a lower glycemic potential than their regular counterparts. The present study would seem in support of the general hypothesis that inclusion of fibre is beneficial for improved blood glucose control, keeping in mind that other differences than pea fibre-enrichment might contribute to explain our findings. 

Surprisingly, inclusion of 9% fat in the pea fibre-enriched bread did not alter the glycemic response. Previous studies have suggested that fat may modify the rate of glucose absorption by delaying gastric emptying [39]. However, this study did not aim at exploring the different mechanisms involved in the observed effects.

It may be difficult to find objective measures of satiety. Many studies have been performed in order to explore the effect of delayed gastric emptying and rapid changes in blood glucose levels and hormones on satiety. In the present study we used a simple questionnaire to compare the subjective feelings of satiety after ingestion of three standardized bread meals. From our study, increasing the percentage of pea fibre had an effect both upon peak postprandial glucose levels and satiety.

All the subjects in this study had a waist circumference ≥80 cm, which render, it possible that these subjects suffer from metabolic syndrome [40]. Since we did not include determination of fasting triglycerides and HDL in this study, we are not able to evaluate if the participants fall into the definition of the metabolic syndrome. Also the lack of insulin data represents a limitation of the present study. It is, however, conceivable that attenuation of the postprandial glucose response would be reflected in a lower insulin response as well.

Great care was taken to ensure that equal quantities of available carbohydrates were ingested in the test meals. However, there were different CHO sources in the different breads. The design of this study is not appropriate to evaluate the different effects of each particular ingredient, and this represents a limitation of this study. 

In affluent countries, a major part of the day is spent in the postprandial phase in which people have their highest blood glucose levels. Hence, it is of special interest to focus upon strategies to reduce high postmeal blood glucose levels. In the present work we have demonstrated appreciable beneficial effects of increasing the pea fibre content in bread. Regular use of pea fibre-enriched bread could be beneficial for attenuating high postprandial excursions of blood glucose, thereby possibly being beneficial for subjects with reduced glucose tolerance. Regular use of pea fibre-enriched bread could be beneficial for weight reduction due to high satiety ratings in combination with attenuated blood glucose levels and lower calorific content if people eat the same amount of bread. Further studies are, however, required to elucidate whether this diet alteration might also serve to prevent type 2 diabetes and cardiovascular diseases. We emphasize that this study did not aim at comparing the effects of different kinds of fibre. Studies on long-term effects on other coronary risk factors of replacing traditional bread with pea fibre-enriched ones are currently in progress.

## 5. Conclusions

Breads containing a great percentage of pea fibre seem to blunt the rise in blood glucose while still keeping the satiety at a high level, but the apparent effects might partially be attributed to other components. No effect seems to be obtained by increasing the content of rapeseed oil in the pea fibre-enriched bread.

## Figures and Tables

**Table 1:** Baseline characteristics of the participants.

	Mean	SD
Age (years)	46	7.9
BMI (kg/m^2^)	31.0	5.0
Waist circumference (cm)	96.8	12.2
Blood pressure (systolic/diastolic) (mmHg)	120/82	16.7/10.9
Fasting blood glucose (mmol/L)*	5.6	0.5
Fasting blood glucose (mmol/L)**	5.6	0.2
2-h blood glucose value (mmol/L)** (OGTT)	8.3	1.9

*Mean value of 9 measurements (3 measurements each of the 3 experimental days).

**Mean value of 3 measurements performed during the OGTT.

**Table 2:** Portion sizes and contents of bread tested.

	Bread types					
	Bread 1		Bread 2		Bread 3	
	(low fiber, low fat)		(high fiber, low fat)		(high fiber, high fat)	
Portion size						
g bread	61		119		133	
(corresponding g CHO)	(25 g CHO)		(25 g CHO)		(25 g CHO)	
Content	Per100 g	Per portion	Per 100 g	Per portion	Per 100 g	Per portion
Energy (kcal)	220	**135**	145	**173**	200	**266**
Dietary fibre (g)	4.6	**2.8**	17	**20.2**	17	**22.6**
Protein (g)	8.7	**5.3**	12	**14.3**	11	**14**
Carbohydrate minus fibre (g)	40.1	**25**	21	**25**	19	**25**
Fat (g)	2.8	**1.7**	1	**1.2**	9	** 12**

**Table 3:** Various measures of the glycemic response after ingestion of different types of bread.

Glycemic measure	Bread 1	Bread 2	Bread 3
	(low fat, low fiber)	(low fat, high fiber)	(high fat, high fiber)
	25 g CH	25 g CH	25 g CH
PV (mmol/L)	9.2 ± 0.4^a^	8.0 ± 0.2	7.6 ± 0.3
TTP (min)	47 ± 4	48 ± 5	52 ± 4
IPV (mmol/L)	3.6 ± 1.1^a^	2.4 ± 0.7	2.0 ± 0.6
GP (min/mmol/L)	28.9 ± 9.2^a^	48.5 ± 14.0	60.6 ± 18.3
IAUC (mmoL × L^−1^× min)	177.6 ± 23.2	143.1 ± 16.8	134.6 ± 17.4

PV: average postprandial blood glucose peak value; TTP: time to peak value, IPV: incremental peak; GP: and glycemic profile; IAUC: incremental area under the 120 min glucose concentration versus time curve (IAUC).

^a^ *P* < .05 versus Bread 2 and 3.
